# The contribution of pediatric surgery to poverty trajectories in Somaliland

**DOI:** 10.1371/journal.pone.0219974

**Published:** 2019-07-26

**Authors:** Emily R. Smith, Tessa L. Concepcion, Mubarak Mohamed, Shugri Dahir, Edna Adan Ismail, Henry E. Rice, Anirudh Krishna, on behalf of the Global Initiative for Children’s Surgery

**Affiliations:** 1 Duke Global Health Institute, Duke University, Durham, NC, United States of America; 2 Department of Public Health, Robbins College of Health and Human Services, Baylor University, Waco, TX, United States of America; 3 Edna Adan University Hospital, Hargeisa, Somaliland; 4 Sanford School of Public Policy, Duke University, Durham, NC, United States of America; Xiamen University, CHINA

## Abstract

**Background:**

The provision of health care in low-income and middle-income countries (LMICs) is recognized as a significant contributor to economic growth and also impacts individual families at a microeconomic level. The primary goal of our study was to examine the relationship between surgical conditions in children and the poverty trajectories of either falling into or coming out of poverty of families across Somaliland.

**Methods:**

This work used the Surgeons OverSeas Assessment of Surgical Need (SOSAS) tool, a validated household, cross-sectional survey designed to determine the burden of surgical conditions within a community. We collected information on household demographic characteristics, including financial information, and surgical condition history on children younger than 16 years of age. To assess poverty trajectories over time, we measured household assets using the Stages of Progress framework.

**Results:**

We found there were substantial fluxes in poverty across Somaliland over the study period. We confirmed our study hypothesis and found that the presence of a surgical condition in a child itself, regardless of whether surgical care was provided, either reduced the chances of moving out of poverty or increased the chances of moving towards poverty.

**Conclusion:**

Our study shows that the presence of a surgical condition in a child is a *strong singular predictor* of poverty descent rather than upward mobility, suggesting that this stressor can limit the capacity of a family to improve its economic status. Our findings further support many existing macroeconomic and microeconomic analyses that surgical care in LMICs offers financial risk protection against impoverishment.

## Introduction

The provision of health care in low-income and middle-income countries (LMICs) is increasingly recognized as a significant contributor to economic growth. Many areas of surgical care are highly cost-effective, with similar values to traditional public health programs.[1–4] The World Health Organization (WHO), the World Bank, and the United Nations have all noted that access to adequate surgical care is essential to achieve the Sustainable Development Goals (SDGs), particularly towards poverty reduction.[5–7] Surgery is essential to both health-system strengthening and universal health coverage, as a functional health system cannot be realized without surgical access.[1, 8, 9]

The provision of surgical care in LMICs not only affects national economies on a macroeconomic level, but also impacts individual families and communities at a microeconomic level. By macroeconomic measures, the failure to provide surgical care has been estimated to lead to reduction in gross domestic product (GDP) by as much as 2% in many LMICs by 2030.[1] Most existing microeconomic studies have measured the financial burden of medical costs on impoverishment, with up to 81 million people estimated to be at risk of falling into poverty due to catastrophic expenditures related to direct costs of surgical care as well as indirect costs of required lodging, transportation, and food.[1] However, these studies do not measure how the presence of a surgical condition itself impacts the risks of poverty, regardless of health care expenditures. Furthermore, given that up to 50% of the population in the majority of LMICs are children and many of these children will have surgical conditions,[10–20] the financial impact of surgical care for children constitutes a much-needed research area.[21] Why some families are devastated by surgical costs while similar families are able to rebound economically is not well understood.[22]

The trajectories of families either falling into poverty or coming out of poverty over time in LMICs may offer a helpful measure of how health conditions and medical costs affect household poverty. Most existing health economic analyses require comparison of expenditures with family income, which is challenging in many low income countries where a high percentage of the population depend on subsistence income.[22, 23] In contrast, the Stages of Progress method is a community-based, validated, and widely used methodology to accurately assess poverty status and fluxes in LMICs. These research tools are not dependent on measure of annual income, rather they are based on assessment of family’s acquisitions and depletions of assets over time. The Stages of Progress scale assesses the structural conditions of households, and limits stochastic or transient variations.[24] These scales can be used to identify factors which affect poverty trajectories, which is critical for policy development.[22, 25]

Somaliland is a low-income country in Sub-Saharan Africa and the 4^th^ poorest country in the world, with tremendous economic and health care challenges as it rebuilds from civil conflict.[26] Our previous work has identified a high burden of surgical conditions in children across Somaliland, as well as need for expansion of surgical infrastructure and workforce, particularly in rural areas.[27] The primary goal of our current study was to examine the relationship between surgical conditions in children and the poverty trajectories of families across Somaliland. We hypothesized that some families would face severe poverty descents by either the presence of a surgical condition itself or surgical care for their children.

## Methods

### Setting

Our study took place in Somaliland, a country in the Horn of Africa which although not recognized as an independent state, has achieved relative stability since separation from Somalia with an autonomous government since 1991. The country has a GDP per capita of $347, classifying it as a low-income country by World Bank income groups and the fourth poorest in the world.[28] Approximately 53% of the population lives in urban centers, 11% in rural areas, and 34% is nomadic or semi-nomadic.[28] Infant and under-5 mortality rates are 91 and 72 per 100,000 respectively,[29] compared to the overall mortality rates in Sub-Saharan Africa of 55 and 83 per 100,000, respectively.[30, 31] Somaliland includes 6 regions: Awdal, Maroodi Jeex, Sahil, Sanaag, Sool, and Togdheer. Approximately 50% of the total population of 4 million people are children under the age of 16.[32]

### Participants and data collection

Our current study was an extension of previous work examining the burden of surgical conditions in children as well as hospital infrastructure and workforce in Somaliland.[27] We collected data between August and December 2017 from 838 households across all regions of Somaliland for a total of 1,503 children, described in detail elsewhere.[27] In brief, we used community-based sampling of survey clusters in all regions of Somaliland. Survey clusters were randomly selected in a two-stage process with a probability adjustment for population by region and cluster. Random selection was country-wide through proportional-to-size methodology and participants were note randomly selected based on the presence of a surgical condition, income level, or any other demographic factor. This work used the Surgeons OverSeas Assessment of Surgical Need (SOSAS) tool, a validated cluster-based, cross-sectional survey designed to determine the burden of surgical conditions within a community.[33–37] We collected information on household demographic characteristics, including household size and financial information, and surgical condition history on children younger than 16 years of age.

We defined surgical conditions using the Lancet Commission on Global Surgery (LCoGS) as “any disease, illness, or injury in which surgical care can potentially improve the outcome.”^1^ A surgical need was self-reported by the parents or guardians of the children as a condition that required surgical consultation. The respondents were first asked if their child has had a wound, burn, mass / goiter, deformity (congenital or acquired) or problem with specific problems associated with a certain body region. If so, follow up questions were asked about problem specifics, surgical treatment sought, and disability. Prior to data analysis, conditions were confirmed as surgical by one pediatric surgeon not involved with data collection. The lifetime prevalence of surgical conditions was determined as the rate of children who reported a surgical condition at some point in their life. Respondents were asked if any type of care was provided for these surgical conditions, including care provided at a healthcare facility (defined as care provided by a physician or a nurse at a health facility) or traditional care (defined as care provided by a traditional healer outside of a healthcare facility).

#### Poverty trajectories

To assess poverty trajectories over time, we measured household assets using the Stages of Progress framework (see detailed description[25, 38]). Using concepts from the Social Capital Assessment Tool (SCAT),[39] we queried each household for the assets they owned at different points in time. Stages of progress from poverty to non-poverty included asset acquisitions of chickens, farm animals, TV/radio, refrigerator, washing machines, and cars. Reference time point cutoffs were used, including over the past three years (as referenced when the current drought started in Somaliland) and seven years (as referenced by the previous Somaliland presidential election). We calculated the total number of household’s assets and defined the accumulation or loss of assets over time as an indicator of financial stability and poverty trajectory in line with the Stages of Progress framework.[22, 25]

**Fig 1:**
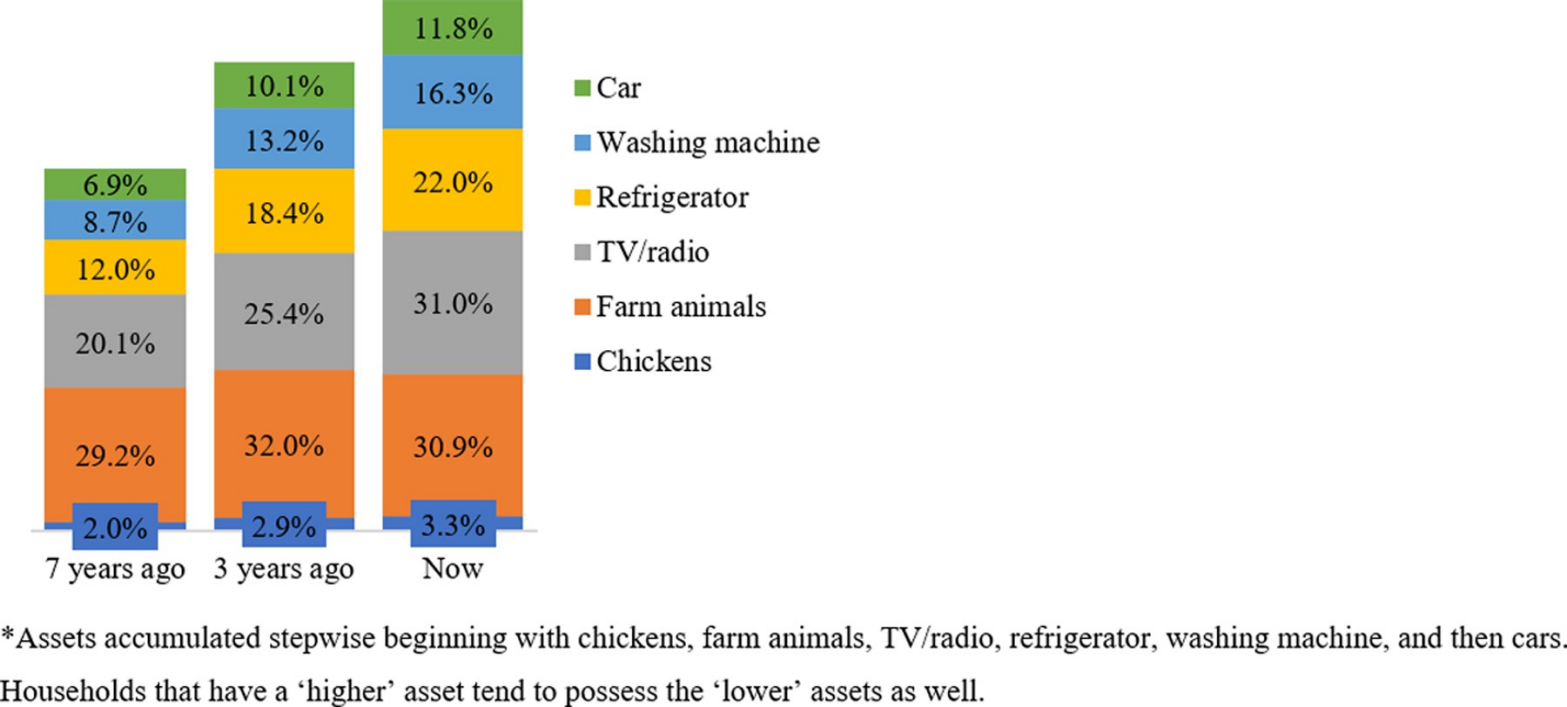
Descriptive status of household assets in Somaliland.

Given that household income is difficult to accurately measure for many households in LMICs as many do not work in the formal sector or have a consistent income,[22, 23] we used assets as an indicator of poverty status.[24] We calculated an asset score for each household by summing the total of assets. This construction was justified because, as we found, assets are usually accumulated in a step-wise fashion, with functional assets (such as chickens and ducks) accumulated first and expensive or luxury assets (such as refrigerators, washing machine and cars) accumulated later–in sequence, as illustrated later (see Fig 1).[38]

### Data analysis

Data on total assets was collected at the time of survey (denoted current, 2017), three years prior to survey (2014), and seven years prior to survey (2010). Therefore, two intervals were recorded to measure changes in assets: between seven to three years prior to survey, and between three years prior to survey and current. Families were identified as **moving away from poverty** (“Moved up”) if their interval changes (from 7–3 years, from 3-years-current) were zero/positive, positive/positive, positive/zero, or negative/positive, with the second change being the most recent. Families were identified as **moving towards poverty** (“Moved down”) if their interval changes were zero/negative, negative/negative, negative/zero, or positive/negative. Families were identified as **remaining neutral towards poverty** (“Stayed same”) if interval changes were zero/zero (**Table 1**).

**Table 1:** Poverty trajectory identification from changes in asset totals.

CHANGE IN ASSET TOTAL			
Between 3 years– 7 years ago	Between now– 3 years ago	No.	DIRECTION
Increased	Increased	34	Moved up
No change	Increased	88	
Increased	No change	111	
Decreased	Increased	11	
No change	No change	492	Stayed same
Decreased	Decreased	17	Moved down
Decreased	Stayed the same	47	
Stayed the same	Decreased	25	
Increased	Decreased	13	

Households and individuals were weighted based on known regional populations from census data[40] and pediatric proportion estimates.[32] Mean asset scores were compared between groups using the weighted values and categorical variables were compared using the weighted chi-square statistic.

#### Multivariate models

To identify variables associated with poverty trajectories, we used multivariate logistic regression analysis using a logit model. Variables used in the multivariate models were chosen based on univariate associations with the outcome and included demographic characteristics (household income, household size, region, presence of a pediatric surgical condition, and receipt of surgical procedure). Odds ratios and confidence intervals were calculated while adjusting for other covariates. Data were analyzed using SAS 9.4 (SAS, Cary, NC) and Microsoft Excel 2010 (Microsoft, Redmond, WA). All data were analyzed incorporating proportional-to-size methodology, cluster-based sampling, and design weights based on sampling fractions.

### Ethical consideration

Institutional Review Board (IRB) approval was granted from the Duke University Institutional Review Board. Since Somaliland does not have a national IRB, a letter of approval was granted from the Somaliland Ministry of Health. Participants in the community survey offered verbal consent for study participation. A parent or guardian provided consent for all children younger than 15 years old, and children between the ages of 12 and 15 provided assent. For the majority of children enrolled, parents answered all questions in the survey.

## Results

We found there were substantial fluxes in poverty across Somaliland over the study period, although overall most families moved away from poverty over the past seven years as the country rebounds from recent civil conflict. We confirmed our study hypothesis and found that the presence of a surgical condition in a child itself, regardless of whether surgical care was provided, either *reduced* the chances of moving *out* of poverty or *increased* the chances of moving *towards* poverty.

### Demographic and asset profile

Over half of the household members were 15 years of age or younger, with a mean household size of 6.7 members, and approximately 50% of households were from rural regions (**Table 2**). We found no statistically significant differences in household demographics when stratified by poverty trajectory status. Among the 838 households, 21.2% (n = 178) reported having a child with a surgical condition (**Table 3)**. Families with a child with a surgical condition were more likely to have a greater number of family members (p = 0.004), more children (p = 0.003), reside in more urban regions such as Maroodi Jeex (p = 0.047) and to have a monthly household income of less than $100 (p = 0.075) compared to families of children without surgical conditions.

**Table 2:** Household demographics, stratified by poverty trajectory status (N = 838).

	Total (n = 838)	Down (n = 102)	Same (n = 492)	Up (n = 244)	p
**Village Type**					
Rural	51.0 (399)	64.7 (64)	58 (272)	27.8 (63)	0.2885
Urban	49.0 (439)	35.3 (38)	42 (220)	72.2 (181)	
**Household size**	6.7 (0.2)	6.5 (0.3)	6.6 (0.2)	7 (0.2)	0.2971
**No. children** **per household**	3.4 (0.1)	3.4 (0.3)	3.5 (0.1)	3.4 (0.1)	0.649
**Household age**					
< 1 y	2.4 (146)	1.7 (11)	2.5 (92)	2.7 (43)	0.1695
1–5 y	18.9 (1090)	19.8 (138)	19.6 (654)	17.2 (296)	
6–10 y	18.2 (1002)	17.6 (125)	18.9 (593)	16.8 (279)	
11–15 y	14.1 (759)	14.7 (92)	14.1 (441)	13.9 (225)	
> 15 y	46.3 (2623)	46.2 (301)	44.9 (1479)	49.4 (834)	
**Household income (monthly, all sources)**					
$0 - $100	34.5 (271)	57.7 (52)	37.7 (186)	15.9 (33)	0.1365
$100 - $400	32.9 (272)	20 (25)	34 (155)	36.5 (92)	
$400 or more	7 (53)	5 (4)	6.4 (27)	9.3 (22)	
Unknown or missing	25.6 (242)	17.3 (21)	21.9 (124)	38.3 (97)	
**Region**					
Awdal	16.4 (90)	14.7 (14)	20.9 (66)	6.5 (10)	0.1288
Maroodi Jeex	40.7 (488)	27.3 (46)	32.4 (252)	66.9 (190)	
Sahil	2.8 (40)	1.7 (3)	3.1 (27)	2.7 (10)	
Sanaag	13.1 (60)	17.4 (10)	13.5 (38)	10.1 (12)	
Sool	8.7 (39)	7.1 (4)	11.6 (32)	2.5 (3)	
Togdheer	18.3 (121)	31.7 (25)	18.5 (77)	11.2 (19)	

**Table 3:** Household demographics, stratified by households with a child with surgical conditions (N = 838).

Characteristic	TOTAL (n = 838)	NSC (n = 660)	CSC (n = 178)	p
**Village Type**	**% (n)**	**% (n)**	**% (n)**	
Rural	51.0 (399)	53.1 (325)	42.6 (74)	0.182
Urban	49.0 (439)	46.9 (335)	57.4 (104)	
Household size*	6.7 (0.2)	6.5 (0.2)	7.4 (0.4)	0.004
**No. children** per household*	3.4 (0.1)	3.3 (0.1)	3.9 (0.1)	0.003
**Household age**				
< 1 y	2.4 (146)	2.4 (107)	2.7 (39)	0.316
1–5 y	18.9 (1090)	18.8 (842)	19.3 (248)	
6–10 y	18.2 (1002)	17.8 (751)	19.4 (251)	
11–15 y	14.1 (759)	14 (579)	14.4 (180)	
> 15 y	46.3 (2623)	46.9 (2026)	44.3 (597)	
**Household income (monthly, all sources)**				
$0 - $100	46.4 (271)	44.0 (205)	56.4 (66)	0.214
$100 - $400	44.2 (272)	46.2 (224)	35.7 (48)	
$400 or more	9.4 (53)	9.8 (45)	7.8 (8)	
**Region**				
Awdal	16.4 (90)	17.3 (73)	13 (17)	0.047
Maroodi Jeex	40.7 (488)	39 (376)	47.7 (112)	
Sahil	2.8 (40)	2.4 (27)	4.6 (13)	
Sanaag	13.1 (60)	14.3 (52)	8.5 (8)	
Sool	8.7 (39)	7.8 (28)	12.3 (11)	
Togdheer	18.3 (121)	19.3 (104)	14 (17)	

NSC: Households without children with surgical conditions; CSC: Households with children with surgical conditions.

*mean (SE)

### Poverty status and trajectories

Between 2010 and 2017, most homes across Somaliland moved away from poverty as measured by an increased total number of assets (**Fig 1**). The rate of households reporting owning a refrigerator, washing machine, or car nearly doubled between 2010 and 2017, while the rate of households owning a TV or radio increased by approximately 10%. Currently, approximately one-third of households own farm animals or chickens (34.2%), 31.0% own a TV or radio, 22.0% own a refrigerator, 16.3% own a washing machine, and 11.8% own a car. Three years prior to survey, there were a slightly lower number of assets, with 34.9% of households reporting farm animals or chickens, 25.4% a TV or radio, 18.4% a refrigerator, 13.2% a washing machine, and 10.1% a car. Seven years prior to survey there were lower rates of total owned assets, with 31.2% of households reporting owning chickens and farm animals (31.2%), TV or radio (20.1%), refrigerator (12.0%), washing machine (8.7%), and a car (6.7%)

### The association of poverty trajectories and pediatric surgery

**Fig 2:**
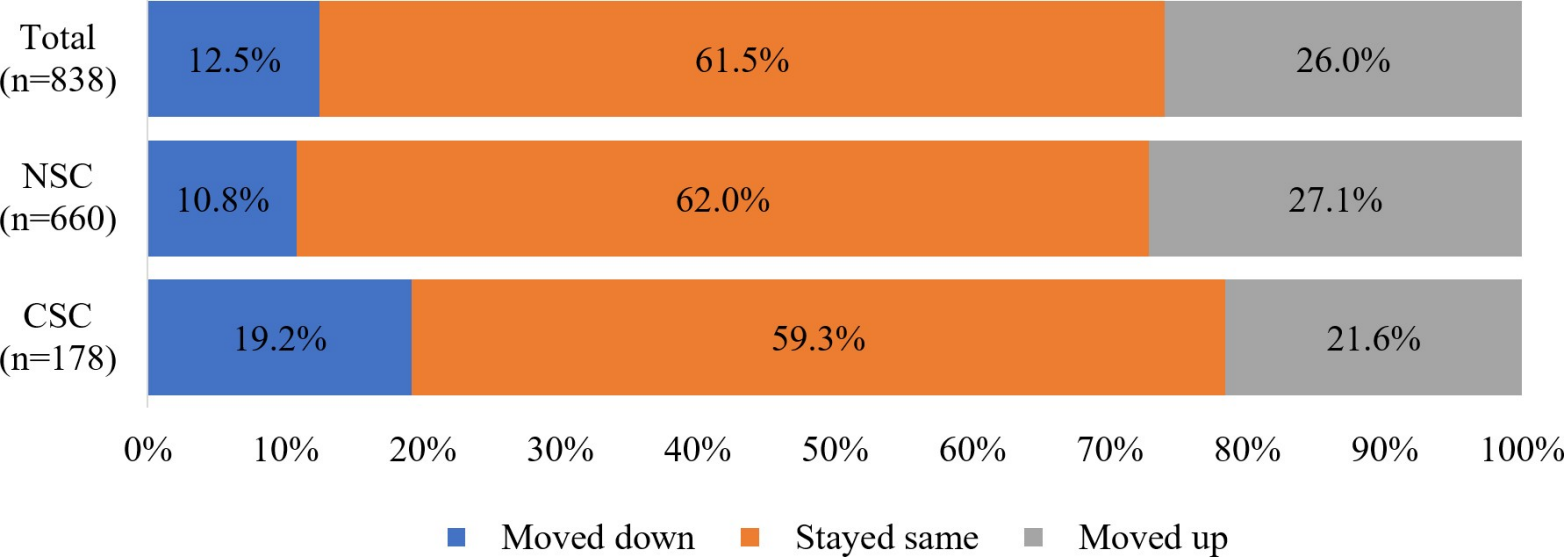
Poverty trajectory of households stratified by presence of child with surgical condition (n = 838).

We found that the presence of a surgical condition in a child, *regardless of whether or not surgical care was received*, led to risk of loss of assets or moving towards poverty for families across Somaliland (**Fig 2)**. We found that overall, a greater proportion of households with a child with surgical conditions (CSC) moved down in assets (19.2%) compared with households without a child with surgical conditions (NSC, 10.8%) (p = 0.05). Current asset scores and changes in assets differed for CSC households depending on whether the child received surgical care (**Table 4**). Current mean asset scores were *higher* among NSC homes (1.1) than among CSC homes with children with surgical conditions if they did not receive surgery, regardless if they sought healthcare (1.0) or not (0.8). However, the current mean scores were *lower* among NCS homes (1.1) than among homes with children with surgical conditions who received surgery (1.8) (p = 0.037). The same trend is seen when asset scores are compared between 2017 and 2010 (p = 0.045) and between 2014 and 2010 (p = 0.022), but not when compared between 2017 and 2014 (p = 0.0147). Current asset scores and changes in asset scores also differed for CSC homes depending on whether the child received surgery. Homes with children with surgical conditions that received surgery had a higher current asset score than homes whose child did not receive surgery (p = 0.037).

**Table 4:** Assets owned and poverty trajectory, stratified by level of healthcare received (n = 1503).

	Household total (n = 838)	No surgical condition (n = 1307)	Surgical conditions (n = 221)			p-value all groups*	p-value surgery (y/n)†
			No healthcare, no surgery (n = 64)	Healthcare, no surgery (n = 95)	Surgery (n = 53)		
**Current asset score**	1.2 (0.3)	1.1 (0.3)	0.8 (0.2)	1.0 (0.3)	1.8 (0.3)	0.021	0.037
**Now and 3 years ago**	0.1 (0.1)	0.1 (0.1)	0.2 (0.1)	0.0 (0.1)	0.2 (0.1)	0.147	0.226
**Now and 7 years ago**	0.3 (0.2)	0.3 (0.2)	0.1 (0.1)	0.2 (0.3)	0.4 (0.3)	0.045	0.020
**3 and 7 years ago**	0.2 (0.1)	0.2 (0.1)	-0.1 (0.1)	0.1 (0.2)	0.3 (0.2)	0.022	0.014

*All group p-value compares children with no surgical conditions, surgical conditions which did not receive healthcare or surgery, surgical conditions which sought healthcare but did not receive surgery, and surgical conditions which received surgery (n = 1503).

†**Surgery p-value** compares children with surgical conditions which did not receive surgery and children with surgical conditions which received surgery (n = 221)

### Predictors of moving towards poverty

**Fig 3:**
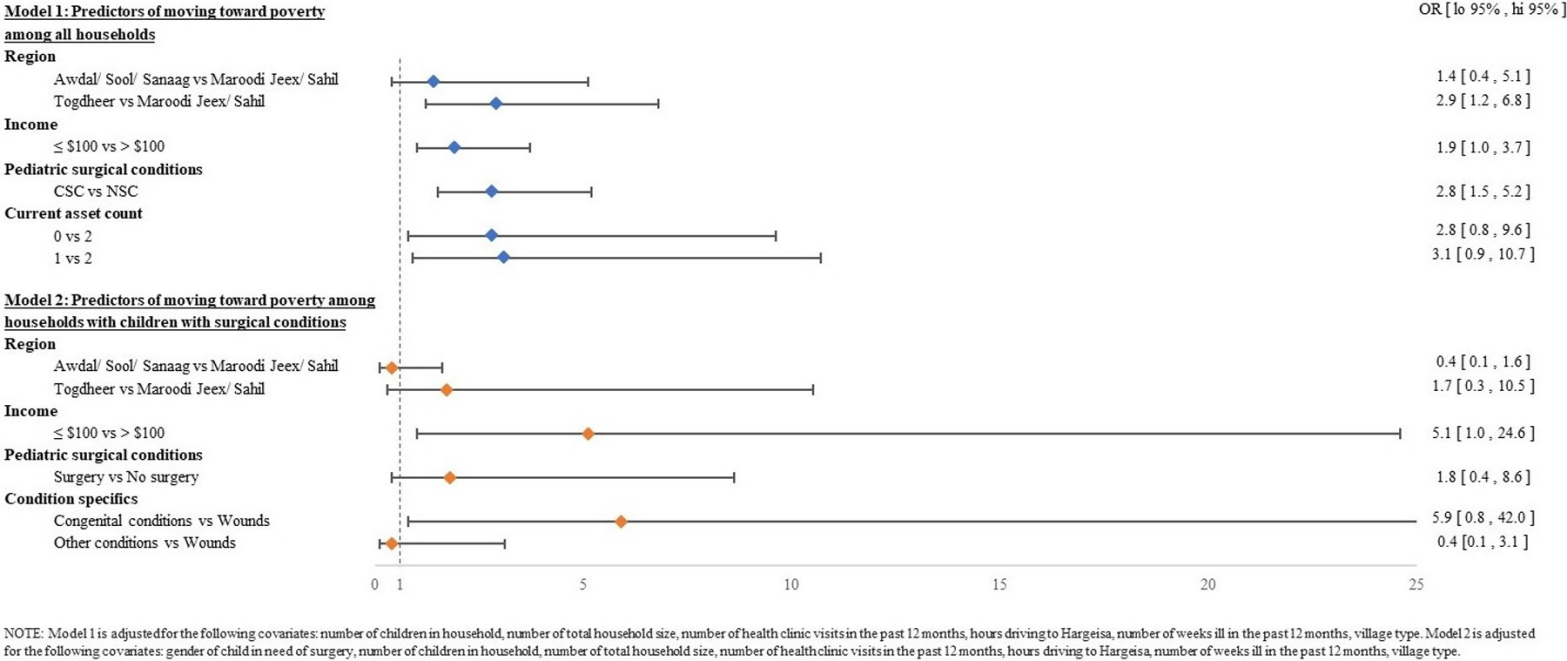
Predictors of moving towards poverty.

Among all households, multivariate analysis confirmed that the presence of a pediatric surgical condition was a significant independent predictor of moving towards poverty (**Fig 3, Model 1)**. The odds of moving towards poverty was 2.8 times higher among CSC homes than among NSC homes (95% CI: 1.5, 5.2; p-value 0.004), adjusting for the other covariates (see full covariate list in **Table 5**). Other significant predictors included the region where the child resided and household income. The odds of moving toward poverty was 2.9 times higher in Togdheer region than Maroodi Jeex (95% CI: 1.2, 6.8; p-value = 0.018), adjusting for other covariates. The odds of moving towards poverty was 1.9 times higher among homes with low incomes of <$100 than homes with higher incomes (95% CI: 1.0, 3.7; p-value = 0.063).

**Table 5:** Full list of covariates in multivariate models.

**MODEL 1: PREDICTORS OF MOVING TOWARDS POVERTY AMONG ALL HOUSEHOLDS**					
	OR	CI		P value	
**Household has a child with a surgical condition (reference NSC)**					
CSC	2.8	1.5	5.2		0.004
**Current asset count (reference 2+)**					
0	2.8	0.8	9.6		0.103
1	3.1	0.9	10.7		0.079
**No. of children (reference 1–3)**					
3+	0.9	0.5	1.4		0.522
**Household size (reference 3–5)**					
6–10	1.9	0.8	4.6		0.166
11+	0.3	0.0	1.8		0.172
**No. of health clinic visits (reference 0)**					
1–3	0.4	0.1	1.9		0.223
4+	0.2	0.0	1.1		0.065
**Hours driving to Hargeisa (reference 0–2)**					
2–6	1.1	0.5	2.4		0.786
6+	1.1	0.2	4.6		0.931
**No. of weeks ill in past year (reference 0)**					
1–2 weeks	5.5	1.7	18.1		0.008
2+ weeks	3.1	0.8	12.6		0.104
**Village type (reference urban)**					
Rural	1.0	0.4	2.1		0.892
**Region (reference Maroodi Jeex/Sahil)**					
Awdal/Sool/Sanaag	1.4	0.4	5.1		0.597
Togdheer	2.9	1.2	6.8		0.018
**Income (reference $100+)**					
$0–100	1.9	1.0	3.7		0.063
**MODEL 2: PREDICTORS OF MOVING TOWARDS POVERTY AMONG HOUSEHOLDS WITH CHILDREN WITH SURGICAL CONDITIONS**					
**Hours driving to Hargeisa (reference 0–2)**					
2+	5.3	1.2	22.8		0.028
**Child gender (reference male)**					
Female	0.6	0.2	2.4		0.496
**Condition specifics (reference wounds)**					
Congenital deformities	5.9	0.8	42.0		0.072
Other conditions	0.4	0.1	3.1		0.390
**Received surgery (reference yes)**					
No	1.8	0.6	5.4		0.279
**No. of children (reference 1–3)**					
4+	1.8	0.4	6.9		0.395
**Household size (reference 3–5)**					
6+	1.8	0.4	6.9		0.395
**No. of health clinic visits (reference 0)**					
1+	0.4	0.0	9.6		0.534
**No. of weeks ill in past year (reference 0)**					
1+	1.8	0.2	20.6		0.619
**Region (reference Maroodi Jeex/Sahil)**					
Awdal/Sool/Sanaag	0.4	0.1	1.6		0.188
Togdheer	1.7	0.3	10.5		0.568
**Village type (reference urban)**					
Rural	1.1	0.4	2.6		0.859
**Income (reference $100+)**					
$0–100	5.1	1.0	24.6		0.045

CI = Confidence interval; CSC = Household with a child with a surgical need; NSC = Household without a child with a surgical need; OR = Odds ratio

Among households with a child with a surgical condition, the odds of moving towards poverty are even more pronounced among the significant predictors (**Fig 3: Model 2**) than among all households in Model 1. Although not statistically significant, the odds of moving towards poverty among CSC homes who received surgery was 1.8 times higher than among CSC homes who did not receive surgery (95% CI: 0.3, 8.6). The odds of moving towards poverty was 1.7 times higher among persons living in Togdheer (95% CI: 0.3, 10.5; p-value 0.0.57) than those living in Maroodi Jeex, adjusting for other covariates. The odds of moving towards poverty was 5.1 times higher among homes with low incomes of <$100 than homes with higher incomes (95% CI: 1.0, 24.6; p-value = 0.045), adjusting for other covariates. Lastly, the odds of moving towards poverty among CSC homes was 5.9 times higher for families with a child with a congenital deformity compared with families with a child with wounds (95% CI: 0.8. 42.0; p-value = 0.072), adjusting for other covariates.

## Discussion

Understanding the association between poverty trajectories and health care in LMICs has tremendous potential for policy development. Since recent civil conflict, most families in Somaliland have had an overall improvement in asset measures and ascent out of poverty. Although these improvements are notable and encouraging for the country, our study shows that economic progress has not been equal among all families. Most notably, the presence of a surgical condition in a child is a strong predictor of poverty descent rather than upward mobility, suggesting that this single stressor can limit the capacity of a family to improve its economic status. The present study supports the original study hypothesis that the presence of a surgical condition in a child, regardless if surgical care was provided, is associated with poverty descent.

Our analysis confirmed previous studies that surgical care can have a devastating economic impact on families in LMICs. Modeling studies from the LCoGS have estimated that up to 33 million individuals face catastrophic health expenditures due to medical costs of surgery, and an additional 48 million facing catastrophic expenditures from non-medical costs of transportation, food, and lodging alone.[1] We have extended these analysis to assessment of the impact of surgical care on family assets, and found that households with a child with a surgical condition were poorer with lower asset scores and were more likely to descend into poverty than homes without a child with a surgical condition.

Interestingly, the financial impact of a surgical condition increased the risk of moving towards poverty regardless whether or not the child received surgical care, although the reasons for these changes are not defined by our analysis. We suspect though for families with limited assets who could not access surgical care for their child, the presence of the condition itself with long-term associated morbidities may have put additional chronic financial strain on the family, detracting them from the capacity to climb out of poverty. For families with assets to access surgical care, both direct and indirect health care costs may have contributed to their descent towards poverty. These findings highlight the importance of incorporating all families with a child with surgical conditions into future research, regardless of whether the family accessed care. Although not all assets a family could have had were asked about, the assets recorded through the questionnaire according to the Stages of Progress scale have been shown to a reliable and valid indicator and proxy for financial stability and income.[24] Most existing macro- and micro-economic research focus only on direct and indirect costs of care, not accounting for the vast majority of patients with surgical conditions who do not receive care at all. Thus, the global financial impact of surgical conditions is likely grossly underestimated, with substantial implications for policy guidance and future estimates of the impact of surgical conditions on global economies.

Families in our study who received surgical care for their child had significantly higher current asset scores than families without a child with a surgical condition. This suggests that the families receiving health care are among the most affluent in Somaliland, consistent with many other LMICs. Our sampling methodology was proportional to population (i.e., weighted) and randomized. Thus, our finding that the most affluent families received surgeries is not a self-selection problem, due to the randomized sampling strategy, but rather a major finding of the study. A recent World Bank survey in Somaliland found that children born into poorer households were less likely to receive medical care that more affluent households.[41] These findings confirm the value of integrating surgical care as part of universal health coverage (UHC). Reducing the costs of a child’s surgical procedure may improve healthcare-seeking behaviors among poor families, thereby potentially improving the family’s overall poverty trajectory. By macroeconomic metrics, Somaliland is among with world’s poorest countries, with 29% of people in urban areas and 38% of those in rural areas classified as living in poverty,[41] ranking the region as the 4^th^ poorest country in terms of GDP per capita. In terms of extreme poverty, the rural/urban disparity is even more pronounced, with 24% of people in rural Somaliland living under extreme poverty, compared to 8% of urban areas.[41]To address risks of poverty descent related to surgical care in Somaliland, implementation of several health financing reforms will likely be required. From a policy standpoint, incorporating systems for the financing of surgical care for children as part of UHC is critical to improve the health care of children. Although such investments can be substantial, as shown in the LCoGS, the required investments to provide basic surgical care are far lower than the estimated financial losses if such reforms are not undertaken.^42^

This study has several limitations. Data was collected through a community-based, household assessment by non-surgeon enumerators. Although the data collectors were medical professionals, they may have not been adequately trained to assess the presence of surgical conditions in the households. Nomadic families and families residing in conflict-prone areas of the upper northeast regions may have been underrepresented. If these families are poorer than the ones in our households, our estimates could be biased towards the null and underestimate the association between poverty, location, and the presence of a pediatric surgical condition. Although nomadic populations were not sought out, these groups in Somaliland often reside in *aqals* in rural villages, contributing to the rural population and these households were included in the sampling strategy. Another limitation was families reported household income in terms of monthly amounts. Data collectors noted that families sometimes had difficulty estimating their monthly income for several reasons: monthly income varied from month to month, agricultural and pastoral workers do not always receive a monthly income and thus the value of their livestock is difficult to estimate, and Somaliland uses both the Somaliland shilling and USD as currency. In addition, we were unable to assess the percent of families under the poverty line in Somaliland, as income data was collected in a categorical fashion rather than continuously. Lastly, the effects of surgical conditions on a family’s assets may be endogenous. However, data regarding assets was collected in a retrospective nature by comparing starting and ending assets throughout a time period, thus greatly lessening the risk of endogeneity.

Our findings further support many existing macroeconomic and microeconomic analyses that surgical care in LMICs offers financial risk protection against impoverishment.[1, 2] Extension of universal health coverage to include support for surgical care may contribute to strengthening other aspects of healthcare.[1, 42, 43] We offer several policy recommendations to minimize the risks of movements towards poverty for families of children with surgical conditions:

- A national health care plan should include strategic support for basic surgical services addressing the health needs of children, as increasing evidence supports investing in surgical services is cost-effective and supports strengthening of the health system[1–3]
- Implementation of a universal health coverage package (UHC) should include care for essential surgical procedures, particularly in rural areas of Somaliland
- Inclusion of the total population in LMICs affected by surgical conditions in future economic analyses to accurately measure the impact of poverty and surgery.
